# Functional and Structural Neuroplasticity Induced by Short-Term Tactile Training Based on Braille Reading

**DOI:** 10.3389/fnins.2016.00460

**Published:** 2016-10-13

**Authors:** Weronika Debowska, Tomasz Wolak, Anna Nowicka, Anna Kozak, Marcin Szwed, Malgorzata Kossut

**Affiliations:** ^1^Laboratory of Neuroplasticity, Nencki Institute of Experimental Biology, Polish Academy of SciencesWarsaw, Poland; ^2^CNS Lab, Nalecz Institute of Biocybernetics and Biomedical Engineering, Polish Academy of SciencesWarsaw, Poland; ^3^Bioimaging Research Center, World Hearing Center, The Institute of Physiology and Pathology of HearingWarsaw, Poland; ^4^Laboratory of Psychophysiology, Nencki Institute of Experimental BiologyWarsaw, Poland; ^5^Department of Psychology, University of Social Sciences and HumanitiesWarsaw, Poland; ^6^Department of Psychology, Jagiellonian UniversityCracow, Poland

**Keywords:** neuroplasticity, primary somatosensory cortex, secondary somatosensory cortex, sighted, Braille, fractional anisotropy

## Abstract

Neuroplastic changes induced by sensory learning have been recognized within the cortices of specific modalities as well as within higher ordered multimodal areas. The interplay between these areas is not fully understood, particularly in the case of somatosensory learning. Here we examined functional and structural changes induced by short-term tactile training based of Braille reading, a task that requires both significant tactile expertise and mapping of tactile input onto multimodal representations. Subjects with normal vision were trained for 3 weeks to read Braille exclusively by touch and scanned before and after training, while performing a same-different discrimination task on Braille characters and meaningless characters. Functional and diffusion-weighted magnetic resonance imaging sequences were used to assess resulting changes. The strongest training-induced effect was found in the primary somatosensory cortex (SI), where we observed bilateral augmentation in activity accompanied by an increase in fractional anisotropy (FA) within the contralateral SI. Increases of white matter fractional anisotropy were also observed in the secondary somatosensory area (SII) and the thalamus. Outside of somatosensory system, changes in both structure and function were found in i.e., the fusiform gyrus, the medial frontal gyri and the inferior parietal lobule. Our results provide evidence for functional remodeling of the somatosensory pathway and higher ordered multimodal brain areas occurring as a result of short-lasting tactile learning, and add to them a novel picture of extensive white matter plasticity.

## Introduction

Neuroplastic changes accompanying the processes of new skill acquisition or associative learning have been studied in both humans and animals. It is well established that following sensory learning, the cortex of the modality specific to the task shows modifications in electrophysiological properties, neuronal responsiveness, synaptic, and functional connectivity, gray matter volume, and the structure of white matter (for review see: de Villers-Sidani and Merzenich, 2011; Kilgard, 2012; Zatorre et al., 2012; Lövdén et al., 2013; Sur et al., 2013; Takeuchi and Izumi, 2013). In the somatosensory domain, tactile sensory discrimination was reported to alter receptive fields and excitability of neurons of the primary somatosensory cortex, and induce representational plasticity in both humans and animals (Recanzone et al., 1992; Siucinska and Kossut, 1996 Pleger et al., 2003; Gebel et al., 2013; Ladda et al., 2014; Andrew et al., 2015). Rapid recruitment of the primary visual cortex for touch after short-term visual deprivation (Merabet et al., 2008) and alterations in activity of supramodal cortical regions have also been shown (Pleger et al., 2003; Saito et al., 2006; Eckhoff et al., 2008; Groussard et al., 2010; Sathian et al., 2013).

Sensory learning was also found to increase neuronal excitability in animal learning models (Saar and Barkai, 2003; Ohl and Scheich, 2005; Matthews and Disterhoft, 2009; Bekisz et al., 2010) and stronger activation may induce increases in myelination (Sampaio-Baptista et al., 2013; Gibson et al., 2014; McKenzie et al., 2014) or axonal sprouting (Beaulieu, 2002; Boele et al., 2013), which may be reflected by its fractional anisotropy (FA). Moreover, only a few functional activation studies were combined with investigation of white matter integrity (Scholz et al., 2009; Taubert et al., 2010; Loui et al., 2011; Gebauer et al., 2012; Schlegel et al., 2012; Lövdén et al., 2013; Draganski et al., 2014; Chavan et al., 2015) and none of them focused on tactile learning.

To address this issue, we investigated the patterns of functional and structural reorganization induced by tactile learning. The learning task was to read Braille exclusively by touch. Our subjects were individuals with normal vision who had no prior experience with the tactile Braille alphabet. Braille reading consists of several distinct processes including simple finger movement, the perception of series of variously arranged raised dots, pattern recognition, and semantic decoding/lexical processing. Learning to read Braille combines mastering the tactile discrimination task with higher cognitive functions. Since the somatosensory modality plays a crucial role in this process, we expected to find changes along the somatosensory pathway including the primary (SI) and secondary somatosensory (SII) cortices, as well as other areas of the ventral somatosensory pathway engaged in processing of tactile information (Burton and Sinclair, 2000; Pleger et al., 2003; Reed et al., 2004).

While use-dependent plasticity within the primary somatosensory representation is well documented, much less attention has been paid to the secondary somatosensory area. The SII is a higher-order, somatotopically organized cortical area known for its participation in tactile and learning processes, decision making, and susceptibility to the influence of attention (Johansen-Berg et al., 2000; Fujiwara et al., 2002; Romo et al., 2002; Sadato et al., 2002; Pleger et al., 2003). It is also considered to be a multimodal area that integrates information from both sides of the body (Huttunen et al., 1996), but its exact role is still unknown. We previously reported bilateral plasticity of the vibrissae representation within the SII of mice resulting from a short-term sensory training, which was the first demonstration of functional changes to this area induced by associative learning in rodents (Debowska et al., 2011). Unilateral structural (voxel based morphometry, VBM) change of the SII cortex associated with tool-use learning was reported in primates by Quallo et al. (2009). Therefore, the second objective of our study was to understand the relative contributions of the somatosensory cortices to learning by determining whether the secondary somatosensory cortex in humans also undergoes neuroplastic changes induced by sensory training.

Besides somatosensory areas, we expected to observe training-induced changes within multisensory cortical regions known to be involved in tactile recognition: the fusiform gyrus/lateral occipital cortex and associative parietal areas (Amedi et al., 2001, 2002; Sadato et al., 2002; Kassuba et al., 2011; Kim and Zatorre, 2011; Marangon et al., 2016).

## Methods

In the present study, a group of sighted individuals underwent two scanning sessions (fMRI and DTI) before and after they learned to read Braille exclusively by touch, without any visual deprivation (subjects also kept their eyes open during trainings and MRI sessions).

### Participants

21 right-handed and sighted subjects (11 women, 10 men, min. age = 22, max. age = 26, mean age = 24.1, *SD* = 1.4) with no history of neurological or psychiatric disorders participated in the study (Oldfield, 1971). All participants gave their written informed consent prior to the start of the experiment and received financial compensation for completed participation. None of the subjects had prior experience with Braille reading. The study was approved by the Ethics Committee of the Warsaw Medical University.

### Experimental procedure—braille training

Training consisted of 15 Braille reading lessons (45 min per day) conducted over the course of 3 weeks. The Braille teaching procedure was planned under the supervision of the Polish Association of the Blind. A Braille 36-chapter ABC-book printed on plastic with standard Braille dimensions (Marburg Medium) was used for the purpose of learning to read Braille (Andraszewski, 2006). As the ABC-book is designed for use by visually-impaired people, it enables us to explore and learn subsequent Braille characters exclusively by touch. The new skill of Braille character recognition was acquired by tactile association with embossed (same-size dots) letters of the Latin alphabet. The first couple of chapters introduced the Braille alphabet with the subsequent ones providing exercises and readings solely in Braille. The six-dot cell (rectangles) used in a further part of the experiment for non-Braille tactile recognition (control) did not appear during the training, so this particular spatial dot-pattern remained untrained and ascribed no particular meaning. The greatest emphasis of the training was put on practice, such that the first lesson included reading a series of up to six-letter words. Subjects were trained to use only their index finger of the right hand while learning, while the left hand was used to guide the right hand position on the text. Participants kept their eyes open during the trainings and the Braille text was concealed by a partition. Additionally, they were asked not to look up the visual Braille displays. Trainings were conducted in small groups (3–4 people) by the same teacher. Great care was taken to teach each subject in the same manner and to a similar skill level. The first nine chapters introduced letters, so during trainings subjects repeatedly returned to them throughout the entire course. After completing the basic lessons, subjects were trained in reading short words and then short sentences that comprised short stories. Since reading skill was balanced among the group, subjects were able to progress up to the 30th chapter over 15 sessions.

### Brain imaging

The first brain imaging session was conducted 1 day before beginning the trainings and the second session 1 day after the last training.

### fMRI—braille character discrimination task

**Figure 1:**
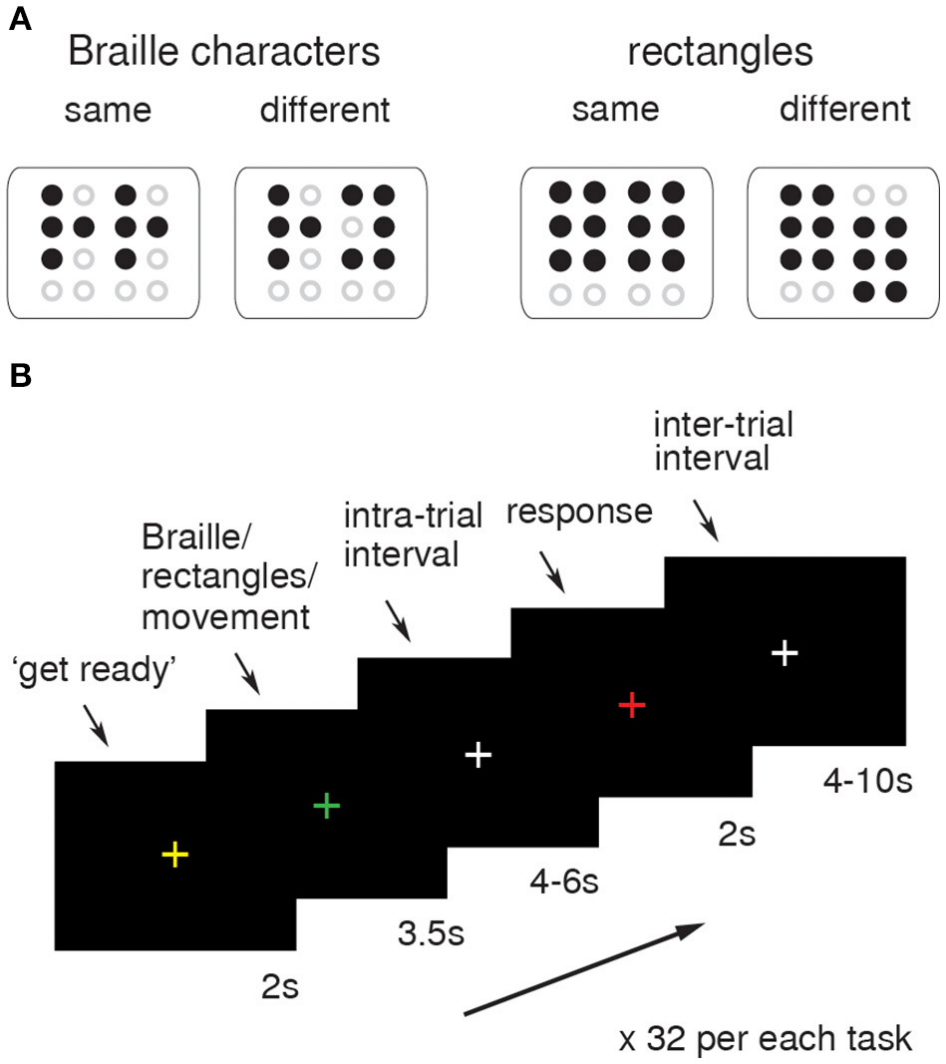
**fMRI procedure**. Examples of same-different Braille characters and rectangles used as tactile stimuli during functional run **(A)**. The schema of the visual presentation used as a task-cue for subjects. The change of the fixation cross (“+”) was coupled with tactile (Braille/rectangles) stimuli presentation and submission of answers **(B)**.

During the functional imaging sessions, the subjects were asked to actively explore and compare two simultaneously presented Braille characters or non-Braille signs (six-dot rectangles; Figure 1A), or simply to move their finger as though they were touching the presented stimuli, but without touching the matrix (control task). The stimuli were perceived by an active touch of the “trained” finger. A total of 32 pairs of Braille characters (chosen from all of the letters of the alphabet, half of which were the same) and 32 pairs of either parallel or shifted positioned rectangles were presented pseudorandomly alternating with 32 simple movement trials, with a constraint of no more than three consecutive trials of the same type in a row. The functional data were obtained using an MRI-compatible Braille Character Stimulator, fully computer-controlled, phneumatically driven, and capable of delivery up to three standard Braille characters simultaneously (2 × 3, Marburg Medium) with addition of delivery also diacritics and meaningless characters i.e., rectangles (for detailes see: Debowska et al., 2013). The stimulator was placed on the subject's thigh, so the arm laid naturally along the body and the “reading” finger was positioned as when reading regular text. Subjects were requested to look at a fixation cross (“+”) located in the center of a black screen during the whole session. Change of the fixation point color was used as a cue. The sequence of colors in a single experimental trial was as follows: starting with white (basic color), a yellow “+” is presented for 2 s as a preparatory cue. The “+” then turns green and the stimuli are presented for 3.5 s, after which it turns back to white. After a few seconds it turns red to signal time for a response and then reverts back to white. In the “movement” condition trials, conducted in order to identify brain activations associated with finger movement *per se*, the preparatory cue was presented in blue. Intra- and inter-trial intervals varied across tasks and were administered randomly for 4 to 6 s and 4 to 10 s, respectively. Examples of tactile stimuli and a single trial sequence schema are presented in Figures 1A,B respectively. The delay between the stimulation and the response cues was introduced in order to obtain “pure” activation from unilateral stimulation. Subjects submitted their answers by pushing the corresponding button on a ResponsePad® (SMIT-LAB, www.smit-lab.eu) with their left hand. The software package PRESENTATION® (Neurobehavioural Systems, Albany, CA) was used to present stimuli and register subjects' responses. The stimuli application procedure used for the active tactile discrimination task in this study guaranteed precise (Braille) stimuli size, resolution, and presentation timing.

### MRI acquisition

All images were acquired on a 3T MRI scanner (Magnetom Trio TIM VB17, Siemens Healthcare, Germany) using a 12-channel head matrix coil. All subjects underwent two identical scanning sessions: before and after the Braille training. Detailed anatomical, functional, and diffusion-weighted images were acquired during both sessions.

### fMRI data acquisition and analysis

**Figure 2:**
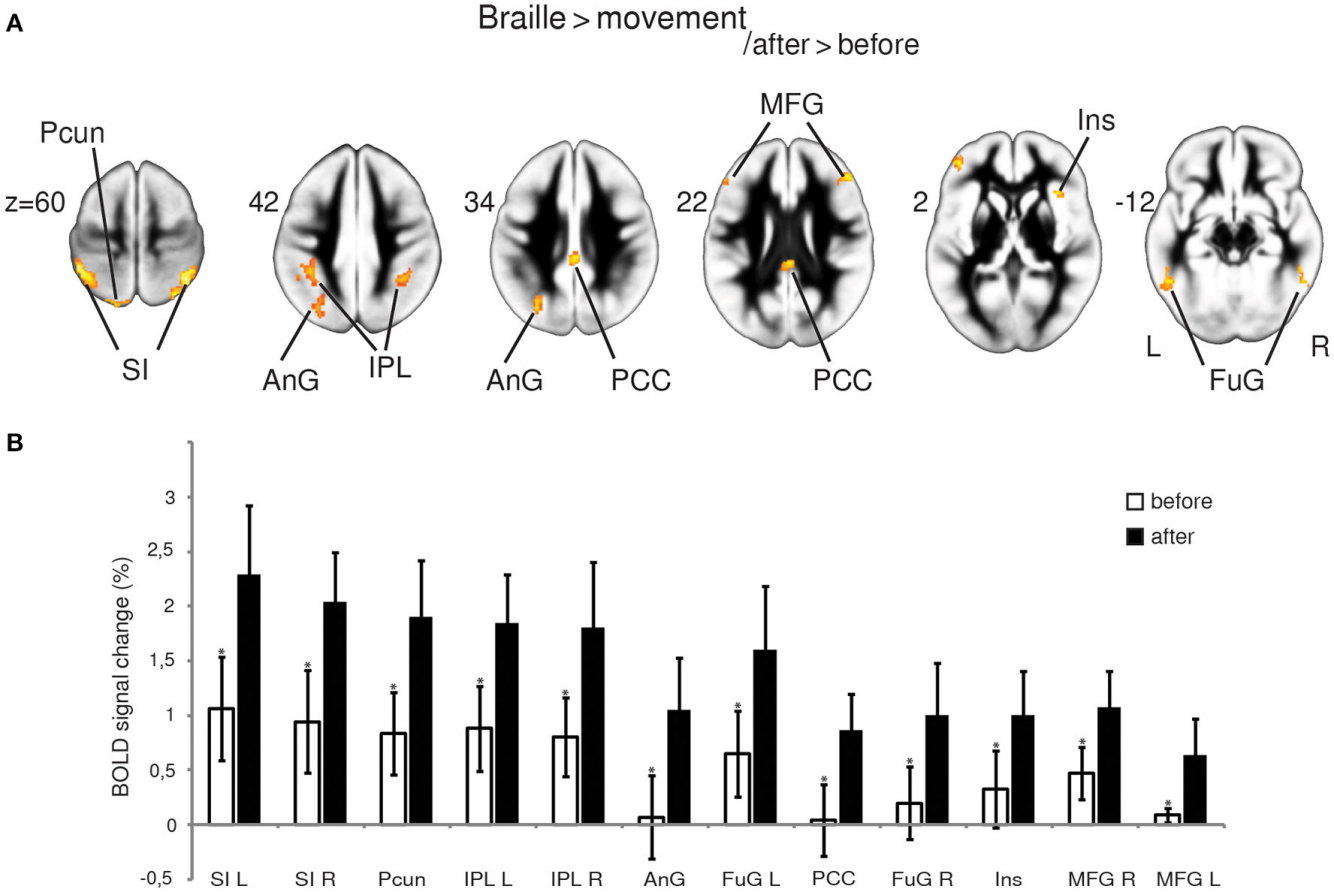
**fMRI results**. Statistical parametric maps of the functional plasticity induced by Braille training revealed by Braille > movement after vs. before training comparison, FWE corrected at *p* < 0.05 **(A)**; percentage of BOLD signal change **(B)**. Percentage of signal change was calculated individually within a spherical volume of interest with a diameter of 10 mm (*p < 0.05, paired *t*-test). Abbreviations: AnG—angular gyrus, FuG—fusiform gyrus, Ins—insula, IPL—inferior parietal lobule, MFG—medial frontal gyrus, PCC—posterior cingulate cortex, Pcun—precuneus, SI—primary somatosensory cortex, L—left, R—right.

**Figure 3:**
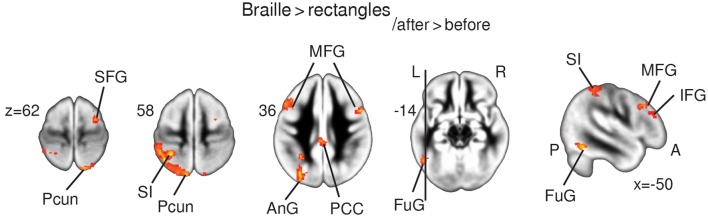
**fMRI results**. Statistical parametric maps of functional plasticity induced by Braille training. Results from Braille > rectangles _after > before_ comparisons are presented on the axial slices of the gray matter template (MNI). FWE corrected at *p* < 0.05. Abbreviations: AnG—angular gyrus, FuG—fusiform gyrus, IFG—inferior frontal gyrus, Ins—insula, LinG—lingual gyrus, MFG—medial frontal gyrus, PCC—posterior cingulate cortex, Pcun—precuneus, SFG—superior frontal gyrus, SI—primary somatosensory cortex.

Anatomical data (T1-weighted) were obtained with 1 mm isotropic resolution (TR = 1900 ms/TE = 2.2 ms/FA = 9 deg./FOV = 192 mm/IPAT = 2/TA = 22:11 min, 47 axial slices perpendicular to AC-PC line). In the functional run a total of 440 volumes (plus 5 dummy scans) were obtained during Echo-Planar Imaging sequences with the following parameters: TR = 3000 ms/TE = 30 ms/voxel: 2 × 2 × 3 mm/FA = 90 deg. SPM12 (http://www.fil.ion.ucl.ac.uk/spm/) was used for data preprocessing and analysis. First, functional images were motion corrected. Then, structural images from single subjects were co-registered to the mean functional image. In the next step, T1 images were warped to the MNI (Montreal Neurological Institute) T1 image template (voxel size of 2 × 2 × 2 mm), and normalization parameters were applied to the functional images, wherein these data were smoothed using a 5-mm isotropic Gaussian kernel. A high-pass filter with a 128 s cutoff was applied to remove low-frequency fluctuations in the BOLD response. For each subject and for each time period separately (before and after training), the onsets and stimuli durations were entered into the design matrix and modeled in a general linear model according to different event types. Regressors were convolved with the canonical hemodynamic response function (Friston et al., 1995). Specific condition effects were assessed by the application of linear contrasts, where parameter estimates for events (i.e., discrimination of Braille characters) were compared to the movement condition. Overall, there were four planned contrasts of interest at the first level of analyses: Braille vs. movement and Braille vs. rectangles each before and after the training. At the second level (whole-brain), paired *t*-tests were used: Braille > movement _after > before_ and Braille > rectangles _after > before_. The statistical threshold was set at *p* < 0.001 and corrected to *p* < 0.05 for multiple comparisons (Family Wise Error) at the cluster-level using cluster size. Detailed results of statistical analysis with MNI coordinates for peaks, clusters size, and exact values used as an extent thresholds are presented in Table 1. Statistical parametric maps for appropriate comparisons superimposed on the gray matter template (GM tissue probability map) are presented in Figures 2, 3. Percentage of signal change was calculated individually within a spherical volume of interest with a diameter of 10 mm using Marsbar (Brett et al., 2002) and then compared using paired *t*-test.

**Table 1:** **Regions showing significant increases in activation as a result of the Braille training (Braille discrimination vs. control tasks × session interaction)**.

***k* = 95**	***x***	***y***	***z***		**Cluster**	***T* (peak)**
**BRAILLE > MOVEMENT/_AFTER > BEFORE_**						
Postcentral Gyrus (SI)	−40	−52	60	L	576	7.17
	−42	−50	56			6.84
Inferior Parietal Lobule	−44	−36	46			7.51
	−36	−42	44			6.58
Angular Gyrus	−28	−74	40	L	332	6.16
	−28	−64	46			5.53
Precuneus	−22	−76	56			4.59
Postcentral Gyrus (SI)	40	−56	58	R	215	7.18
	44	−50	60			5.69
Posterior Cingulate Cortex	0	−34	32	L/R	208	5.83
	2	−32	22			5.37
Middle Frontal Gyrus	−44	46	4	L	176	5.73
	−46	38	14			5.30
	−50	34	20			4.75
Middle Frontal Gyrus	50	36	22	R	151	5.67
	44	34	28			5.40
Inferior Parietal Lobule	36	−48	42	R	122	5.48
	42	−42	40			4.66
Fusiform Gyrus	−50	−52	−16	L	102	6.91
Fusiform Gyrus	56	−54	−14	R	100	5.31
	56	−42	−10			5.02
Insula	38	20	2	R	95	5.72
	46	18	−6			4.42
***k*** = **78**						
**BRAILLE > RECTANGLES/_AFTER > BEFORE_**						
Postcentral Gyrus (SI)	−38	−56	58	L	1392	9.85
	−42	−50	60			7.32
Angular Gyrus	−32	−84	38			7.19
	−32	−72	42			6.89
Inferior Parietal Lobule	−32	−48	56			6.59
Precuneus	−18	−72	52			5.48
	−20	−68	54			5.3
Middle Frontal Gyrus	−40	10	30	L	399	6.79
Inferior Frontal Gyrus	−42	26	34			6.09
	−42	28	26			6.09
Posterior Cingulate	−2	−38	24	L/R	190	5.76
	−2	−30	36			5.65
	6	−24	24			4.50
Fusiform Gyrus	−50	−56	−18	L	100	7.19
	−40	−52	−16			3.94
Middle Frontal Gyrus	30	−2	64	R	93	6.11
Superior Frontal Gyrus	30	−2	64	R	93	6.11
	28	8	58			4.75
Precuneus	16	−70	62	R	78	5.86
	26	−68	60			5.45

T-values of the peak activations, p < 0.05 (correction for multiple comparisons at cluster level, Family Wise Error), and extent threshold k for an appropriate comparison is indicated in the table. MNI coordinates, peak activation, and volume are given along with the significance threshold estimate. L-left, R-right hemisphere.

### DTI protocol and analysis

Spin-echo diffusion weighted echo planar imaging (DW-EPI) sequence was performed with an isotropic (2 × 2 × 2 mm) resolution, *b*-value of 1000 s/mm^2^, 64 gradient directions and repeated twice in order to increase signal-to-noise ratio. The eddy distortion correction and diffusion tensor parameter estimation were performed using ExploreDTI (Leemans et al., 2009). Fractional anisotropies (FA) were calculated for each subject before and after training. SPM12 was used for motion correction, normalization procedures including co-registration to the FA template, smoothing using 3-mm isotropic Gaussian kernel algorithm, and statistical analysis employing paired *t*-tests (Abe et al., 2010). The statistical threshold was set at *p* < 0.001 and corrected to *p* < 0.05 for multiple comparisons (Family Wise Error, FWE) at the cluster-level using cluster size. Detailed results of statistical analysis with MNI coordinates for peaks, clusters size, percentage of FA change, and the exact values used as extent thresholds are presented in Table 2. Statistical parametric maps superimposed on the white matter template (WM tissue probability map) are presented in **Figure 5**. Anatomical localization of cortical and white matter regions was verified on the basis of the MNI Space Utility and confirmed manually using the Atlas of the Human Brain and the MRI Atlas of Human White Matter (Mai et al., 2007; Mori et al., 2010).

**Table 2:** **Changes in FA maps after Braille training**.

***k* = 26**	***x***	***y***	**I**		**cluster**	***T***	**%**
Parietal Operculum WM (SII)	−42	−32	26	L	204	7.15	+1.7
	−40	−32	16	L		6.93	
	−40	−16	20	L		6.80	
Postcentral Gyrus WM (SI)	−36	−28	46	L	126	6.28	+2.3
	−38	−36	60	L		5.81	
	−44	−30	56	L		4.89	
Lingual Gyrus WM	16	−84	−10	R	93	7.50	+1.61
	26	−92	−14	R		7.43	
Superior Frontal Gyrus WM	12	12	70	R	85	5.88	+1.65
	14	12	62	R		5.42	
Lingual Gyrus WM	−26	−84	−4	L	76	9.27	+1.8
	−34	−86	−10	L		7.40	
Parahippocampal Gyrus WM	−28	−32	−18	L	75	5.27	+1.5
	−34	−44	−26	L		5.20	
Fusiform Gyrus WM	28	−54	−8	R	62	8.04	+1.1
Middle Frontal Gyrus WM	42	0	58	R	49	6.71	+1.2
	34	−4	62	R		4.67	
Superior Frontal Gyrus WM	−10	−8	56	L	36	9.61	+2.25
	−16	−12	64	L		4.08	
Inferior Frontal Gyrus WM	46	22	−6	R	36	5.87	+1.51
Lingual Gyrus WM	−14	−52	0	L	34	6.25	+1.42
Superior Frontal Gyrus WM	−24	8	70	L	30	5.68	+1.3
	−16	12	72	L		5.35	
Thalamus LP	−14	−22	10	L	27	6.99	+1.8
	−16	−24	18	L		4.58	

MNI coordinates, peak activation, and volume percentage of FA change are given along with the significance threshold estimate. Results revealed by paired t-test between the FA maps before and after the training. SI—primary somatosensory cortex, SII—secondary somatosensory cortex, LP—lateral-posterior; L—left, R—right hemisphere. T-values of the peak activations, p < 0.05 (correction for multiple comparisons at cluster level, Family Wise Error), and extent threshold k for an appropriate comparison is indicated in the table.

## Results

### Behavioral results

#### Braille discrimination (same-different) task

Task performance (percentage of correct responses) differed significantly from the first to the second imaging session. A paired *t*-test (*t* = 31.46, *df* = 20) revealed a significant improvement from 37.5% (min. = 34.3, max. = 37.5, *SD* = 4.4) to 91.85% (min. = 90.6, max. = 96.8, *SD* = 6.3) in the Braille character discrimination task performance (*p* < 0.001).

#### Rectangles discrimination task

Comparisons of non-Braille discrimination task performance before and after a posteriori did not reveal significant differences (*t* = 2.13, *df* = 20). In the first session the mean percent of correct responses was 56.9% (min. = 53.1, max. = 68.7, *SD* = 7.6), while in the second session it reached 61.4% (min. = 46.8, max. = 75, *SD* = 8.2).

### fMRI results

We analyzed brain activation patterns accompanying discrimination of Braille characters performed by active touch, before and after a short-term intensive tactile training of Braille reading in sighted subjects (*N* = 21). Statistical parametric maps of task-related brain activity patterns before and after the Braille-based tactile training separately are shown in Supplementary Materials. The Braille discrimination vs. finger movement comparison showed a similar pattern of activity across the brain with stronger activations after the training (Figure S1A); comparing Braille vs. rectangles revealed a massive reorganization of cortical activation within the left hemisphere (Figure S1B). Analysis of the interaction between the two imaging sessions (after vs. before) and the experimental conditions revealed a set of brain areas that changed their activation as result of the training (Figures 2, 3). A detailed description is given below and the full set of obtained results along with statistics is given in Table 1.

### The effects of braille-based tactile training

#### Braille characters discrimination task > finger movement _after > before_

By contrasting the brain activity pattern involved in the Braille discrimination task and motion activity without touching (movement), we aimed to depict the cortical underpinnings of Braille-related somatosensory information processing. Comparing the Braille character discrimination task-related activity to the simple finger movement condition showed a wide network of cortical regions involved in this specific activity before and after the training. Our analysis indicated several structures within this network presenting more pronounced responses while performing the Braille character discrimination task after the training. Notably, a bilateral increase in activity of the primary somatosensory representation (SI) was observed. Stronger activations were also found in the precuneus and the angular gyrus located within the left hemisphere, and in the bilateral inferior parietal lobule. Other areas of increased activation were located within posterior cingulate cortex, the middle frontal gyrus, and the fusiform gyrus bilaterally, as well as in the right insula (Figure 2A). The strength of the BOLD signal change among structures revealed by this comparison is shown in Figure 2B.

#### Braille characters discrimination task > rectangles _after > before_

The Braille vs. rectangles comparison was constructed to isolate the specific effect of Braille character processing as a higher order cognitive task, by subtracting the activation by the same finger movement but crucially without the letter-related connotations. Results depicted a stronger response bilaterally within the precuneus, the posterior, and anterior cingulate cortex, as well as the middle and inferior frontal gyri. Left-lateralized changes in BOLD signal were found within the fusiform and angular gyrus, as well as in primary somatosensory finger representation within the postcentral gyrus (Figure 3).

### ROI analysis of parietal operculum/SII

**Figure 4:**
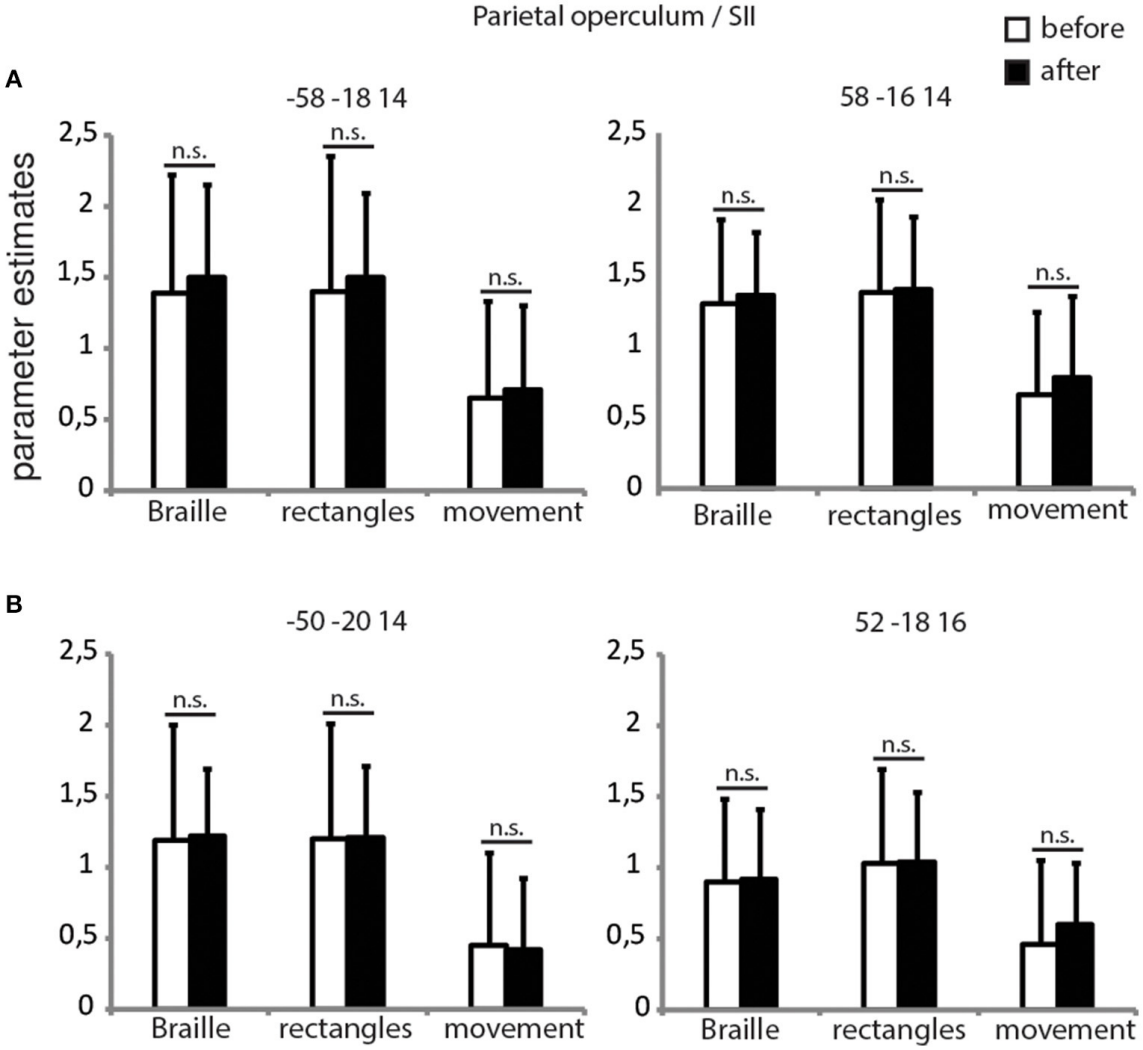
**ROI's for parietal operculum/SII**. Parameter estimates for all of the experimental conditions before and after the training in the secondary somatosensory cortex bilaterally. Coordinates for creating 6 mm spheres were chosen on the basis of our current **(A)** and previous study **(B)**. *p* < 0.05, corrected for multiple comparisons.

Since the whole-brain analysis did not reveal functional changes within the secondary somatosensory cortex, we decided to conduct additional ROI analyses. Two ROI's were created, for each left and right SII, using group data from the Braille discrimination task > finger movement contrast from both sessions (Figure 4A) and coordinates from our previous study (Figure 4B) revealed by the Braille discrimination task > rest comparison (Debowska et al., 2013). Parameter estimates for Braille and rectangles conditions were calculated individually within a spherical region-of-interest with a diameter of 6 mm. Interaction effects were tested using random-effects analysis, with results showing no significant differences in SII activity before and after the training in both the Braille or rectangles discrimination tasks (Figure 4). Based on these results, we conclude that there is no evidence for functional plasticity within the SII attributed to short-term Braille learning.

### DTI results

**Figure 5:**
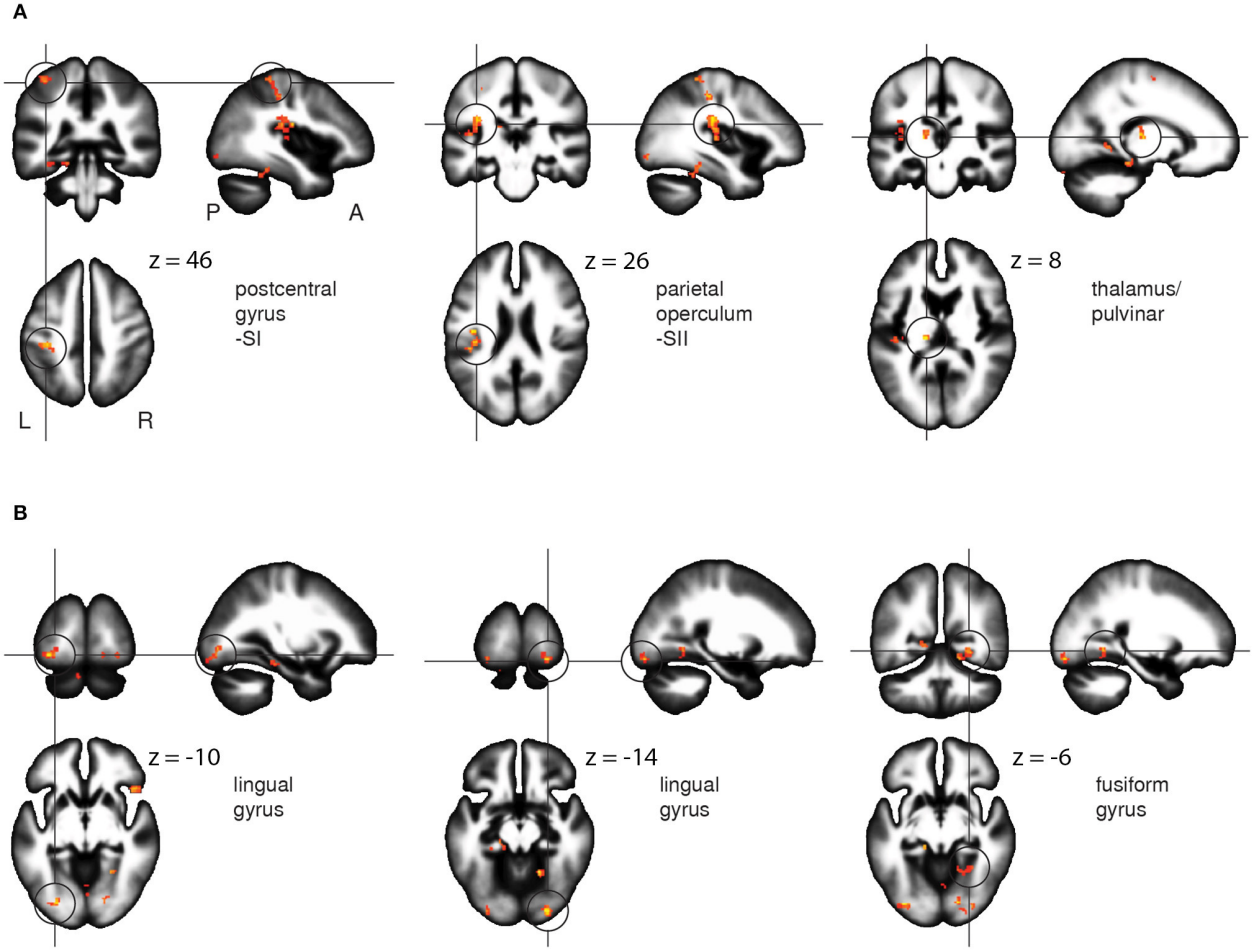
**FA results**. White matter areas showing an increase of fractional anisotropy as a result of the Braille training, within somatosensory (A) and the ventral stream (B) regions. Results revealed by paired *t*-test between the FA maps before and after the training. Statistics presented on the sagittal (x), coronal (y), and axial (z) slices of the white matter tissue template (MNI). FWE corrected at *p* < 0.05, *k* = 26.

Voxel-wise comparison of the FA maps before and after the Braille training revealed a set of the white matter regions in which FA changed as a result of tactile learning. FA was increased among the crucial areas of the somatosensory pathway: the postcentral gyrus (SI), the parietal operculum (SII), and the lateral posterior/pulvinar thalamic nuclei of the left hemisphere (Figure 5A). Other changes were located within frontal and parietal-occipital areas: the right inferior and medial frontal gyri, the superior frontal gyrus, fusiform gyrus, and lingual gyrus bilaterally, and the left parahippocampal gyrus (Figure 5B). The opposite comparisons (before > after) of the FA maps did not reach statistical significance. The full set of obtained results along with the statistics and percentage of FA change is given in Table 2.

## Discussion

Relatively short but intensive tactile training based on Braille reading induced modifications in both primary somatosensory and higher ordered areas. We observed BOLD signal changes likely associated with increased synaptic strength, excitability and expansion in local synaptic connectivity (Saar and Barkai, 2009; Wu and Mel, 2009; Bekisz et al., 2010; Froemke et al., 2013; Jasinska et al., 2013). With DTI, we found increases in the fractional anisotrphy index, likely reflecting strengthening of the intra-cortical connections in response to the demand of increased communication during new-skill acquisition (Blumenfeld-Katzir et al., 2011; Sampaio-Baptista et al., 2004). Changes in fMRI activation were congruent with changes in FA within both somatosensory and inferior occiptiotemporal cortices. The simultaneous use of both methods allows a more precise assessment of task-related plasticity. Its exact impact on relevant brain regions is discussed below.

### Tactile training—the somatosensory pathway

SI. Since Braille reading as a tactile activity must engage somatosensory processing, we expected to find alteration in cortical response and white matter structure within the somatosensory pathway. The contrast of Braille character discrimination vs. finger movement revealed areas that responded stronger to swiping fingers over Braille dots relative to swiping fingers without touching the surface, thus, finger movement acted as a motor control. Following training, the strongest activation increase observed for this contrast was found in the SI (Figures 2A,B) bilaterally. The primary somatosensory cortex was previously reported to manifest mainly contralateral responsiveness (Hari et al., 1993; Deuchert et al., 2002; Hlushchuk and Hari, 2006) and several more recent papers have reported its bilateral involvement in somatosensory information processing (Blatow et al., 2007; Tamè et al., 2012; Chung et al., 2014). The current study reports robust bilateral activation of the primary somatosensory representation during a tactile discrimination task before and—even stronger—after the training. We suggest that this may be a result of increased interhemispheric communication between homotopic regions related to the task complexity and top-down attention (Verstynen et al., 2005; Perez and Cohen, 2008).

In parallel with change in the magnitude of the BOLD signal, we found FA increases within the postcentral gyrus in the region corresponding to the functional localization of the contralateral finger representation within the SI. The increase of activation and in white matter integrity within the SI (Figure 5A) provides strong evidence for the SI's specific engagement in Braille reading skill acquisition. These results are in line with previous findings concerning plasticity of the primary somatosensory representation induced by both short and long-term tactile training (Pascual-Leone and Torres, 1993; Pleger et al., 2003; Hodzic et al., 2004). The increase of white matter integrity around the altered functional SI representation documents structural plasticity as a novel aspect of learning-induced SI modification.

Interestingly, our results are contrary to those recently presented by Sathian et al. (2013), where no effect of tactile learning was found within the SI. We propose that this might be caused by key differences in the experimental procedure—our subjects were trained to actively discriminate different spatial patterns with assigned meaning (Braille characters), subjects in Sathian's study were trained in pure perceptual learning (micro-spatial task based on a linear three-dot array), and the touch was passive. There are also differences in training duration (fixed vs. individual performance criterion), stimuli presentation protocols (active in our study vs. passive) and duration (3 s per pair in event-related design vs. 1 s per single stimulus presented in block-design).

Our results are also divergent from those of Siuda-Krzywicka et al. (2016) who trained sighted subjects in whole-word tactile reading for 9 months. After such a long period of training, Siuda-Krzywicka et al. (2016) did not find changes in the somatosensory cortex. Instead, their results showed an increase in the left fusiform gyrus activation (Visual Word Form Area, VWFA), where most likely this divergence is due to the very long learning period (9 months vs. 3 weeks) and the complexity of stimuli used (whole words vs. pairs of Braille characters). Experiments that study learning-related plasticity at multiple time points (Lövdén et al., 2013) suggest that at the initial stage of Braille learning described by Siuda-Krzywicka et al. (2016), the somatosensory cortex might have increased its response to Braille words, and as the effects of early sensory learning consolidated in the somatosensory cortex, the cortical focus of learning might have shifted elsewhere—to the ventral visual stream.

### Parietal operculum/SII

We found no functional changes in either ipsi- or contralateral SII, but a highly significant FA increase within white matter in the parietal operculum (anatomical localization of the SII) contralateral to the reading finger (Eickhoff et al., 2010). The changes in white matter structure clearly suggest functional involvement of the contralateral SII in acquisition of the new tactile ability, whereas the absence of increased functional activation after 3 weeks of training raises questions regarding the precise character of this involvement. The importance of the SII in tactile processing, including Braille characters' discrimination in sighted individuals (Sadato et al., 2002), is well documented and we see its strong activation during the Braille character discrimination task used in the present study (Figure 4). We propose that lack of the increase in activation of the SII following training might be due to the rigid timeframe used for training. In other words, we speculate that if checked at different (shorter) time points, e.g., a few days after onset of training, the SII might demonstrate its plasticity in terms of functional involvement—stronger at the beginning of training and coming back to normal when the skill became easier (Pleger et al., 2003; Vahdat et al., 2014) and when the white matter tracts are already remodeled. An alternative interpretation is that SII is not involved into neuroplasticity indcued by training, but only play its usual role in ‘transmitting’ information to brain regions involved in stimuli processing.

Additionally, the lack of long-term functional change in SII while increased activity was observed in the SI cortex might inform an ongoing discussion about hierarchical vs. parallel information processing between the SI and SII (Chung et al., 2014; Klinger et al., 2015). While early animal studies suggested strict dependencies between the SI and SII cortices by demonstrating the extinction of the SII activity after lesioned within SII (Pons et al., 1988), it was later proposed that SII may process sensory information independently (Zhang et al., 2001). In humans, effective connectivity studies showed information flow relating to texture, from the SI to the parietal operculum (Sathian et al., 2011). Here, if increased activity in the SI does not alter the activity within the SII, it is most likely that the SII reacts independently and is not a simple reflection of the SI. Another possible explanation is that although the SII is necessary in tactile stimuli processing, its role in the well-trained tactile pattern discrimination task is no longer needed because of the altered global brain pattern of activity and stronger engagement of regions specified in the object recognition—FuG (Figure 2; Table 1). The FA increase observed within areas of the parietal operculum might then reflect an enhanced intra-cortical communication occurring while mastering the new tactile recognition task.

### Outside of the somatosensory domain: fronto-parietal cortex and the ventral stream

As Braille characters become meaningful to the subjects as a result of training, it should be possible to determine loci where this meaning is extracted. Although the task in the scanner required only a same/different answer, activation of the brain was strongly affected by the meaning of the discriminanda. When we computed the interaction of Braille > rectangles with the difference between the before- and after- training scans, we found activations in the left fusiform cortex, medial and inferior frontal gyri, and the angular gyrus (Figure 3, Table 1). Increased activation of those areas after training might be language related, since they were found at locations typical to those found in readers of a variety of different scripts (Perfetti et al., 2010; Szwed et al., 2014). Increased activity within ventral stream activation suggest that even short-term tactile Braille reading training may result in activation of brain regions involved in categories recognition (Tyler et al., 2013), which might be interpreted as a having semantic conotations. Alternatively, they might be task-related. Unfortunately, we do not have more appropriate behavioral nor imaging data to discriminate between those two.

The increase in activity within the fusiform gyrus (Figure 3, Table 1) is congruent with previously reported involvement of the lateral occipital cortex/fusiform gyrus in tactile object recognition in both blind and sighted subjects (Sadato et al., 2002; Amedi et al., 2003; Striem-Amit et al., 2012) or multisensory object perception (Kassuba et al., 2011, 2014; Schlaffke et al., 2015). Alternatively, it could reflect the emergence of tactile Braille recognition mechanisms similar to those found in the VWFA (Price and Devlin, 2001; Cohen et al., 2002; Dehaene and Cohen, 2011), recently found in sighted whole-word tactile Braille readers (Siuda-Krzywicka et al., 2016). Interestingly, these fMRI results were accompanied in our study by FA increase in the lingual gyrus bilaterally and within the fusiform gyri (Figure 2, Table 2). Our results might thus constitute the first report of white matter integrity changes within higher-ordered and visual areas induced by short-term tactile training in sighted subjects. Apparently even short-term tactile Braille reading training may result in activation of brain regions involved in language in general and specifically—in reading.

Finally, some of our results (FuG, AnG, PCC, Pcun) have much in common with those observed after Morse code deciphering training using auditory stimuli (Schmidt-Wilcke et al., 2010). Our results not only support these findings, but also complement them with a novel aspect, showing significant white matter changes throughout the network involved in acquisition and usage of alphabet signs perceived by a different (somatosensory) sensory modality.

## Conclusion

Short-term tactile training based on Braille reading increased SI activation, confirming the presence of learning-induced plasticity in the primary somatosensory cortex. No functional effects were found within secondary somatosensory cortex, however FA increase within the parietal operculum suggest that the SII was involved in acquisition of this new sensory skill, and an fMRI measurement at a third time point might have captured a change of BOLD activity also in the SII. Increased activation of the fusiform gyrus followed by structural alteration in white matter of occipital regions demonstrates that a relatively short tactile training can induce plastic changes in the ventral visual cortex. In summary, our study shows how acquisition and mastering tactile discrimination tasks induce modifications in the pattern and intensity of brain activation and white matter integrity of somatosensory areas, higher-ordered pathways and in the thalamus.

## Limitations

Our training has been arbitrary set for a fixed period of time. It would be extremely interesting and informative to conduct longitudinal study with multiple scanning time points (week by week or even day by day) as well as follow up study to determine how long the observed changes persists. More detailed imaging sequences such as spectroscopy might also brought better insight in to the neurobiological underpinnings of the plastic changes. Moreover, since whole-brain analyses of diffusion indices are of several limitations including Partial Volume Effects and EPI distortions, and requires perfect co-registration (Beaulieu, 2002), other analyses such as tractography combined with i.e., computational anatomy (Draganski et al., 2014) would shed some more light on the exact localization of the reorganized tissue and allow for a better insight into nature of neuroplasticity.

## Author contributions

Study conception and design: WD, AN, MS, MK. Acquisition of data: WD, TW, AK. Analysis and interpretation of data: WD, TW, MK. Drafting of manuscript: WD, AN, MK. Critical revision: WD, MS, AN, MK.

### Conflict of interest statement

The authors declare that the research was conducted in the absence of any commercial or financial relationships that could be construed as a potential conflict of interest.

## Supplementary material

Figure S1 **Braille training—overall fMRI results**. Statistical parametric maps of the Braille vs. movement **(A)** and Braille vs. rectangles **(B)** contrasts, separately for before and after the Braille training. Results are presented on a glass-brain in MNI space (view from the top, back, and left of the brain), *p* < 0.05, FWE.
