# Supplementary figures and images for: Functional and Structural Neuroplasticity Induced by Short-Term Tactile Training Based on Braille Reading
Source: Front Neurosci. 2016 Oct 13;10:460. doi: 10.3389/fnins.2016.00460 (PMC5061995; doi:10.3389/fnins.2016.00460)

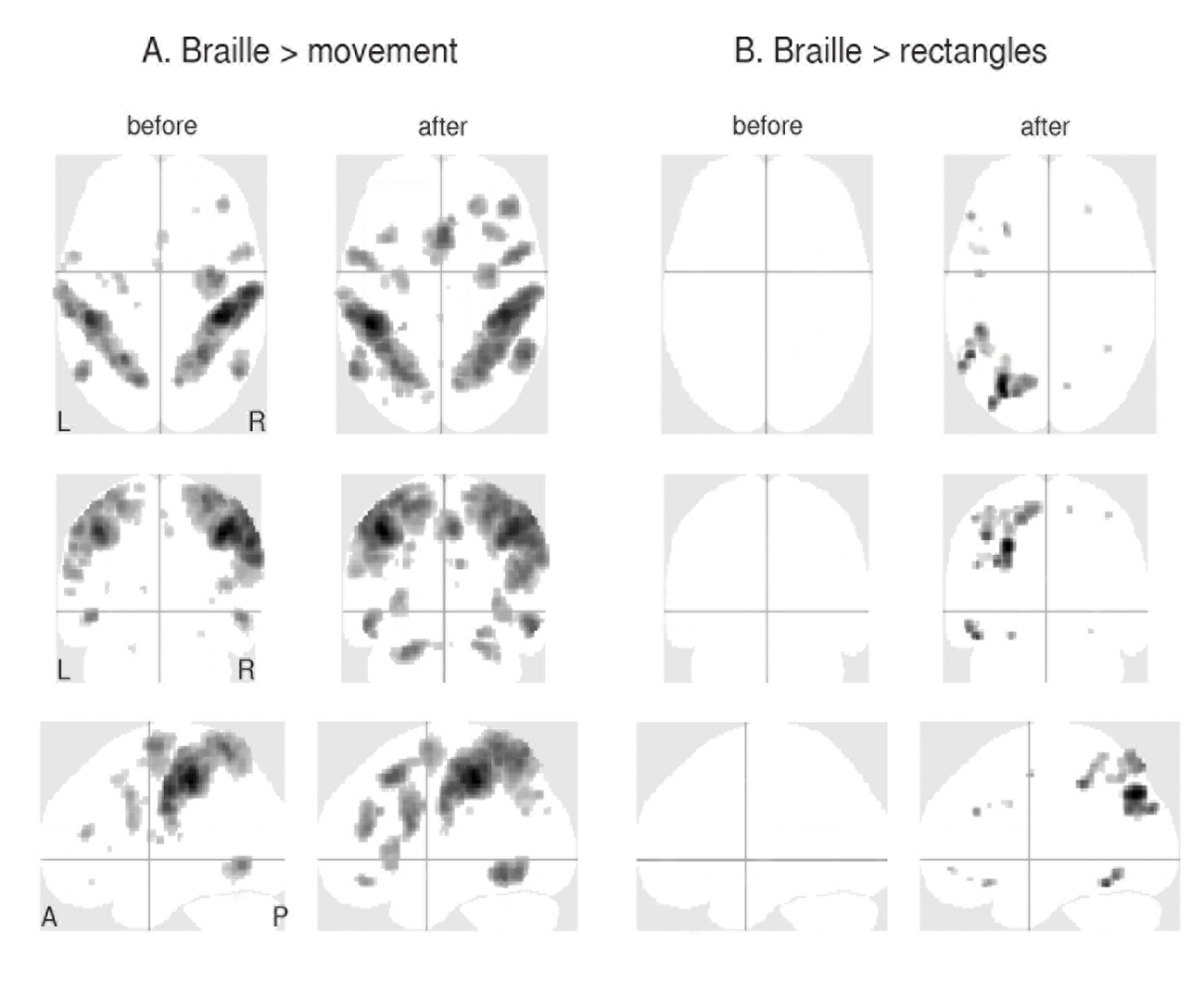

Supplement: Figure S1 — Braille training—overall fMRI results. Statistical parametric maps of the Braille vs. movement (A) and Braille vs. rectangles (B) contrasts, separately for before and after the Braille training. Results are presented on a glass-brain in MNI space (view from the top, back, and left of the brain), p < 0.05, FWE. [file Image1.TIF]
